# A preliminary study assessing cytokine production following ex vivo stimulation of whole blood with diet in dogs with chronic enteropathy

**DOI:** 10.1186/s12917-019-1940-7

**Published:** 2019-06-04

**Authors:** Aarti Kathrani, Edward Hall

**Affiliations:** 1 Present address: Royal Veterinary College, Hawkshead Lane, North Mymms, Hatfield, Hertfordshire AL9 7TA UK; 2 Langford Vets, Langford House, Langford, Bristol BS40 5DU UK

**Keywords:** Canine, Hydrolyzed, Cytokine, Ex vivo, Enteropathy

## Abstract

**Background:**

Ex vivo whole blood stimulation assays (WBSA) have been used to characterize the cytokine response to diet in cats. The present study aimed to use this assay to determine the cytokine response to diets being fed at the time of diagnosis to dogs with chronic enteropathy (CE) and to compare this to a control group of dogs presented for non-gastrointestinal (GI) causes.

**Results:**

Dogs with chronic GI signs and dogs presented for non-GI causes were prospectively recruited. For each case, residual blood following diagnostic sampling was placed into heparin. WBSAs were performed using crude extracts of the diet currently being fed and provided by the owner. Supernatants were collected and analyzed for tumor necrosis factor (TNF)-alpha, interleukin (IL)-10 and IL-4 using an enzyme-linked immunosorbent assay. The case group consisted of 22 dogs with CE diagnosed on histopathology of GI biopsy and 9 with suspected CE. The non-GI group consisted of 18 dogs. Of the diets being fed at or prior to diagnosis, hydrolyzed protein diets elicited significantly lower IL-10 and TNF-alpha concentrations compared to commercial intact protein diets in dogs with confirmed or suspected CE (*P*-value 0.004 and < 0.001, respectively). Six out of 15 dogs with detectable IL-4 concentrations in the confirmed CE group had IL-4 to IL-10 ratios that exceeded the 95% confidence interval (CI) of the mean of the non-GI group (non-GI: 95% CI of IL-4:IL-10 = 0.64–2.71; confirmed CE: IL-4:IL-10 in 6 dogs = mean 22.40, range 2.77–89.11).

**Conclusions:**

Hydrolyzed protein diets elicited a significantly reduced cytokine response when incubated with patient whole blood ex vivo compared to commercial intact protein diets in dogs with CE. The IL-4 to IL-10 ratio as a marker of dietary responsiveness warrants further investigation, together with assessment of the cytokine response to diet at the intestinal mucosal surface.

## Background

Food-responsive enteropathy (FRE) is a sub classification of chronic enteropathy (CE) and describes a group of diseases resulting in chronic gastrointestinal (GI) signs that are responsive to dietary management alone; the underlying etiology of these diseases is unknown [1]. The diagnosis of FRE is made based on the dog’s response to an exclusion diet trial. Dogs with true GI food allergy can present with similar clinical signs to dogs with FRE. Interestingly, studies have shown that intestinal histopathology pre and post treatment with a successful diet trial in dogs with FRE doesn’t change [2, 3], which suggests that the pathogenesis of FRE and food allergy is different. Gastrointestinal food allergy can be subdivided into 2 major categories; immunoglobulin (Ig) E-mediated, which involves mainly mast cells, and non-Ig E mediated, mainly involving T-cells and eosinophils [4]. Unfortunately, intradermal skin testing, skin patch testing and measuring circulating food allergen-specific serum Ig E are of no diagnostic value in GI food allergy due to their low sensitivity and specificity [5, 6]. Therefore food trial and challenge is currently the gold standard for the diagnosis of GI food allergy. Unfortunately, the differentiation of FRE from GI food allergy in dogs is not definitively possible at this time. Although, the return of clinical signs following challenge with the original diet suggests GI food allergy, CE cannot be definitively ruled out as these dogs may also relapse due to individual dietary risk factors.

Similarly, dogs with FRE cannot be definitively differentiated from enteropathy requiring steroid treatment based on history, clinical signs, laboratory parameters or the severity of GI endoscopic or intestinal histological lesions and therefore food trial is currently needed to differentiate the two conditions. As the etiology of FRE is unknown, dogs may respond to different dietary strategies necessitating trials with multiple therapeutic diets before this condition can be ruled out. This may result in frustration and be costly for the owner as well as delay other treatment modalities such as steroids, to which the dog may respond. Therefore, further research is needed to try to differentiate FRE from steroid responsive enteropathy (SRE), as well as from GI food allergy. The ability to differentiate dogs with FRE, SRE and GI food allergy is also important to advance our knowledge of the pathogenesis of individual chronic GI diseases, as this may lead to novel, more efficacious treatments for these separate diseases or change the way we currently treat them. Also, the ability to differentiate FRE from SRE in dogs at the time of diagnosis may help to avoid multiple unnecessary exclusion diet trials.

The author has previously published a study in healthy immunotolerant colony cats using sterile preparations of commercial dry diets to stimulate whole blood ex vivo and measure cytokine production in the supernatant following incubation for 24 h [7]. These stimulation assays used whole blood instead of peripheral blood mononuclear cells (PBMCs) in order to replicate the natural environment of the cells, to prevent changes in phenotype that may occur with isolation of the cells and to minimize the volume of blood needed, as well as the processing time. This methodology using whole blood has been shown to be comparable with the use of PBMCs [8]. The study documented that the majority of cats produced a detectable amount of interleukin (IL)-10 when stimulated with a diet that they had previously tolerated. Interleukin-10 is an anti-inflammatory cytokine and is produced by both the innate and adaptive immune system. Interleukin-10 inhibits cytokines belonging to both the Th1 and Th2 pathways, as well as the expression of autoimmune and pro-inflammatory conditions [9]. One study documented that natural tolerance to foods was associated with increased amounts of IL-10 producing PBMCs in humans [10]. A further study suggested a role for serum IL-10 in the diagnosis of food tolerance in humans [11]. Similarly, the results from the author’s study suggested that increases in IL-10 production in ex vivo whole blood stimulation assays may be used as a marker of tolerance to diet in companion animals [7]. Studies have shown an increase in IL-4 and a decrease in IL-10 concentrations in food-induced GI disorders in humans and mice [12–14]. Interleukin-4 is the signature cytokine for the Th2 immune response and is one of the key cytokines involved in inflammation triggered by allergens [15]. An increase in the pro-inflammatory cytokine TNF-alpha has been shown in cow-milk allergy in children and experimental food allergy in mice [16, 17]. To the authors’ knowledge, no studies have been performed characterizing the cytokine response to food in dogs with CE. Determining the ex vivo whole blood cytokine response to incubated food in these dogs, as well as dogs presented for non-GI causes may help to determine if there is a differential response within dogs with CE and whether this corresponds with the cytokine response seen in food allergy in humans and mice. The results from this study may then lead to further studies to determine if this assay could be used to help sub-classify dogs with CE into FRE, SRE or GI food allergy. Therefore, the aim of our study was to quantify the IL-10, TNF-alpha and IL-4 production following ex vivo incubation of whole blood with diets currently being fed to dogs with confirmed or suspected CE and to dogs presented for non-GI causes.

## Methods

### Dogs

Thirty-five dogs presenting to the University of Bristol Small Animal Referral Hospital between December 1st 2017 and August 31st 2018, with at least a three-week history of intermittent or persistent GI signs were prospectively recruited onto the study. The diagnostic investigations performed for all dogs included a complete blood count, serum biochemistry panel, serum cobalamin and folate concentrations and trans-abdominal ultrasound. The following investigations were also performed in selected cases depending on the history, physical examination and abdominal ultrasound findings: pancreatic lipase immunoreactivity, pancreatic trypsin like immunoreactivity, basal cortisol, pre and post prandial bile acids, fecal parasitology using saturated zinc sulfate flotation, fecal culture for *Salmonella, Campylobacter* and *Clostridium difficile* and collection of intestinal biopsies using endoscopy. Dogs that had intestinal histopathology performed that documented changes consistent with CE were categorized as confirmed CE. Whereas, dogs that had diagnostic investigations that ruled out all known causes of chronic GI signs but did not have intestinal histopathology to confirm CE were categorized as suspected CE. Eighteen dogs presenting for non-GI related causes were also recruited (Table 1).

**Table 1:** Demographics of dogs included in the study; including age, sex, neuter status, breed, diagnosis and diet(s) consumed

		Confirmed CE (*n* = 22)	Suspected CE (*n* = 9)	Non-GI (*n* = 18)
Age (years); median (range)		5 (0.7–10)	3.6 (0.5–11.8)	5.5 (0.5–10.7)
Sex, neuter status	M, MN	4, 6	1,5	7, 2
	F, FN	4, 8	2,1	3,6
Breed (n)		Crossbreeds (7), Labradors (3), Boxers (2), Cocker spaniels (2), Bichon Frise (1), German shepherd dog (1), Lowchen (1), Chihuahua (1), Border collie (1), Cavalier King Charles spaniel (1), Staffordshire bull terrier (1) and Jack Russell terrier (1).	Crossbreeds (3), Cocker spaniel (1), West Highland White terrier (1), Labrador (1), Jack Russell terrier (1), Yorkshire terrier (1) and Springer spaniel (1).	Crossbreeds (5), Miniature Schnauzers (2), German shepherd dogs (2), Cavalier king Charles spaniels (2), Labrador (1), Staffordshire bull terrier (1), Bichon Frise (1), Beagle (1), Husky (1), Whippet (1) and Yorkshire terrier (1).
Diagnosis (n)		Lymphoplasmacytic enteritis (5), lymphoplasmacytic and eosinophilic enteritis (4), eosinophilic enteritis (2), eosinophilic colitis (2), lymphoplasmacytic gastritis (2), granulomatous colitis (1), plasmacytic enteritis (1), lymphoplasmacytic colitis (1), eosinophilic, plasmacytic and mastocytic enteritis (1), lymphocytic colitis (1), plasmacytic colitis (1) and unremarkable partial thickness endoscopic biopsies in a dog with protein-losing enteropathy (1).	Suspected CE (9)	Extra-hepatic portosystemic shunt (3), asymptomatic idiopathic increases in alkaline phosphatase activity (2), sarcoma (2), urethral sphincter mechanism incompetence (1), neutrophilic bronchopneumonia (1), primary hyperparathyroidism (1), suspected hyperadrenocorticism (1), nasal foreign body (1), chronic bronchitis (1), suspected psychogenic polydipsia (1), idiopathic seizure like episodes (1), benign prostatic hyperplasia with possible prostatitis (1), pancreatitis (1) and eosinophilic bronchopneumopathy (1).
Diet(s) consumed	Commercial intact	15	4	16
	Hydrolyzed	4^a^	1	1
	Home-cooked	2	3	1
	Commercial intact + Home-cooked	2	1	0
	Commercial intact + hydrolyzed	1	0	0

Three groups of dogs were included; dogs with confirmed chronic enteropathy (CE; *n* = 22), dogs with suspected CE (*n* = 9) and dogs with non-GI causes (*n* = 18). ^a^ Includes two dogs that had previously been fed a hydrolyzed protein diet, which were also used in the assays for these two dogs, as the owners had provided them at the appointment

Exclusion criteria included dogs that were ultimately diagnosed with or suspected to have GI neoplasia in the GI group, dogs with current signs of dermatological or chronic GI signs in the non-GI group and any dog receiving immunosuppressive doses of corticosteroids in both the GI and non-GI group.

All owners were asked to bring in samples of currently fed diets (10 g of dry or home-prepared food, ready to feed or an unopened tin) to their appointment.

The University of Bristol granted ethical approval for the study (VIN/17/008). Written and oral informed consent was obtained from owners of all dogs that participated in the study.

### Study protocol

#### Preparation of diets for use in ex vivo whole blood stimulation assays

Similar to our previous study, a clean pestle and mortar was used to grind ten grams of the relevant food with sterile water and the mixture transferred to a 50 mL sterile tube^1^ [7]. The sterile tubes containing the mixture were incubated for 2 to 12 h at 4-degrees on a rotator and then centrifuged at 4000 rpm for 12 min at room temperature. The dietary supernatant was then filtered using a 0.22-μm sterile filter^2^ in a sterile tissue culture hood.^3^ The bicinchoninic assay^4^ was used to determine the protein content of the filtrate and a working solution of 500 μg/mL was prepared using sterile phosphate buffered saline^5^ (PBS). This concentration was chosen based on the results of our previous study [7].

For owners feeding a combination of diets, if they were the same diet type (see below) they were processed together resulting in one dietary filtrate, otherwise they were processed separately. Three diet types were used in the study: 1) a commercial intact protein diet, defined as either a commercial over-the-counter diet or a commercial therapeutic diet containing commonly fed ingredients (e.g. chicken, pork, beef or ovine) or a commercial over the counter diet that was labeled to contain less commonly fed ingredients, such as turkey, salmon, venison, trout or duck; 2) a hydrolyzed protein diet, defined as a commercial therapeutic hydrolyzed diet; or 3) a home-prepared diet containing either chicken, pork, salmon, cod, turkey or sardines. Unfortunately, no dogs in the study were being fed a commercial therapeutic limited-ingredient novel protein diet at the time of diagnosis. All commercial therapeutic diets fed in this study were based on European formulations and therefore would be dissimilar to North American formulations of the same diet.

#### Whole blood stimulation assays

For each dog, residual blood from sampling for laboratory testing as part of the diagnostic investigation was placed into heparin and mixed with RPMI^6^ containing penicillin/streptomycin^7^ in a 1:2 volume ratio and 1 mL plated in a 24 well plate^8^ within 1 h of collection. A negative control consisting of 111 μL of PBS^5^ and 500 μg/mL of each diet type consumed by the dog at the time of the appointment was added to separate wells. All samples were analyzed in triplicates for a total of 6 wells for dogs eating one diet type and a total of 9 wells for dogs eating two diet types. Similar to our previous study, all plates were incubated at 37 degrees centigrade in 5% carbon dioxide for 24 h,^9^ after which time they were then spun at 2000 × g for 4 min and the supernatant collected and stored at − 80 **°**C until analyzed [7].

The Canine TNF-alpha Quantikine ELISA Kit^,^^10^ Canine IL-10 Quantikine ELISA Kit^,^^11^ and Canine IL-4 DuoSet ELISA^12^ were used to quantify the concentrations of TNF-alpha, IL-10, and IL-4 in the supernatants according to the manufacturers’ instructions, respectively. The triplicate cell culture wells were assayed individually. A plate reader^13^ was used to measure optical density at 450 nm with a 560 nm wavelength correction within 30 min and the data were analyzed using a computer software.^14^ The lower and upper concentrations of the standard curve of the assays were 15.6 pg/mL and 1000 pg/mL for IL-10, 7.8 pg/mL and 500 pg/mL for TNF-alpha and 93.75 pg/mL and 6000 pg/mL for IL-4. All assays contained a positive control, which was provided by the manufacturer and a negative control, and were performed in duplicate. The standard curve for all plates yielded an R squared value of at least 0.96.

### Data analysis

Similar to our previous study, the mean of each of the duplicate cytokine concentrations was calculated. The mean of these values from the triplicate wells in the ex vivo whole blood stimulation assay was then calculated for the diet and standardized to PBS by subtraction [7].

A One-way analysis of variance (ANOVA) was used to compare IL-10, TNF-alpha and IL-4 concentrations between commercial intact protein, hydrolyzed protein and home-prepared diets in dogs with confirmed or suspected CE. Post hoc analysis was performed using Bonferroni correction. Independent-Samples T-tests were used to compare IL-10, TNF-alpha and IL-4 concentrations, as well as the IL-4:IL-10 and TNF-alpha:IL-10 between dogs with confirmed or suspected CE and dogs presented for non-GI causes. Analyses were performed using IBM SPSS Statistics Version 24. Significance was defined as *P* < 0.05.

## Results

### Demographics of dogs with confirmed or suspected chronic enteropathy and non-GI causes

Thirty-five dogs with chronic GI signs were recruited; 4 were excluded due to confirmed or suspected GI neoplasia. The final case group consisted of 22 dogs with CE diagnosed on histopathology of GI biopsy and nine with suspected CE (Table 1). The non-GI causes group consisted of 18 dogs (Table 1).

### Diet type and ex vivo whole blood stimulation assays in dogs with confirmed or suspected CE

One-way ANOVA comparing the IL-10, TNF-alpha and IL-4 concentrations between hydrolyzed protein diets (*n* = 6), commercial intact protein diets (*n* = 24) and home-prepared diets (n = 6) in dogs with suspected or confirmed CE showed a significant difference for IL-10 and TNF-alpha concentrations, but not IL-4 concentrations (*P*-values 0.005, < 0.001 and 0.125, respectively).

**Fig. 1:**
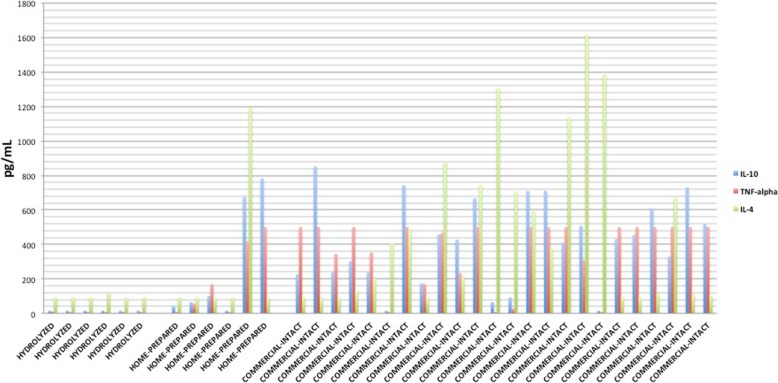
Bar graph showing cytokine production following stimulation of ex vivo whole blood with different diet types in dogs with chronic enteropathy. Bar graph of IL-10, TNF-alpha and IL-4 production following stimulation of ex vivo whole blood with hydrolyzed protein (6 dogs), home-cooked (6 dogs) or commercial intact protein diets (24 dogs) in dogs with confirmed or suspected chronic enteropathy (CE). Hydrolyzed protein diets elicited significantly lower IL-10 and TNF-alpha concentrations compared to commercial intact protein diets in dogs with confirmed or suspected CE (*P*-value 0.004 and < 0.001, respectively)

Post hoc analysis with Bonferroni showed that hydrolyzed protein diets elicited significantly lower IL-10 and TNF-alpha concentrations compared to commercial intact protein diets in dogs with confirmed or suspected CE (IL-10: *P*-value 0.004; mean (standard deviation); hydrolyzed protein diet–15.6 pg/mL (0); commercial intact protein diet – 413.66 pg/mL (248.30), TNF-alpha: *P*-value < 0.001, hydrolyzed protein diet – 8.18 pg/mL (0.94), commercial intact protein diet – 371.88 pg/mL (188.86)). However, there were no significant differences in IL-4 concentrations between hydrolyzed protein diets and commercial intact protein diets (*P*-value = 0.166, hydrolyzed protein diet – 98.36 pg/mL (11.3), commercial intact protein diet – 490.7 pg/mL (473.9)). There were also no significant differences in IL-10, TNF-alpha or IL-4 concentrations between hydrolyzed protein diets and home-prepared diets (*P*-value > 0.220) and commercial intact protein diets and home-prepared diets (*P*-value > 0.105) (Fig. 1).

### Comparison of IL-10, TNF-alpha and IL-4 concentrations between the two groups of dogs

**Fig. 2:**
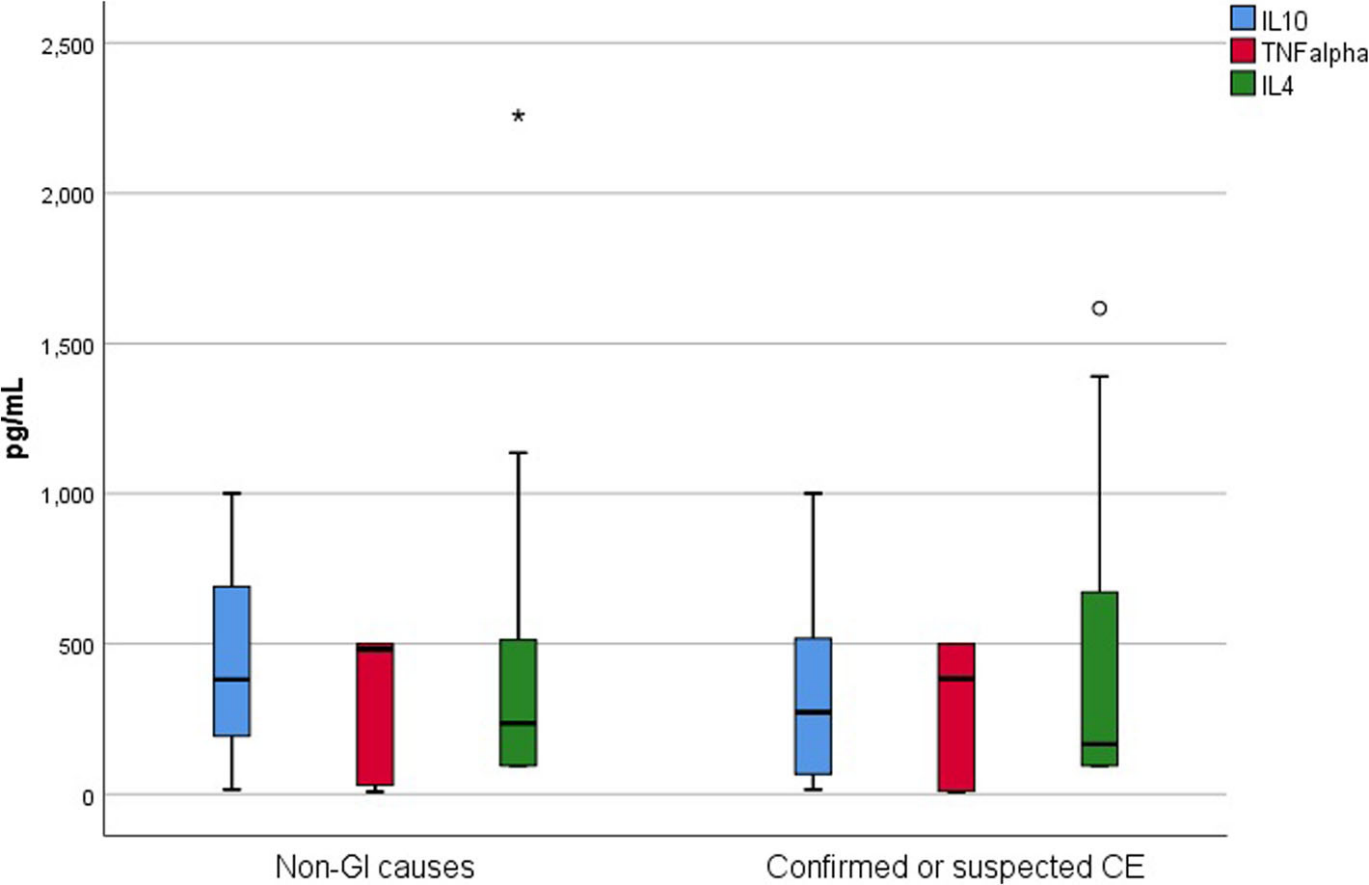
Box and whisker plot showing cytokine production following stimulation of ex vivo whole blood with currently fed diets in dogs with CE and non-GI causes. Box and whisker plot of IL-10, TNF-alpha and IL-4 production following stimulation of ex vivo whole blood with currently fed diets in 18 dogs with non-GI causes and 31 dogs with confirmed or suspected CE. There were no significant differences in IL-10, TNF-alpha or IL-4 concentrations between dogs with confirmed or suspected CE and dogs with non-GI causes (P-value 0.264, 0.858 and 0.834, respectively)

Independent-Samples T-tests showed no significant differences in IL-10, TNF-alpha or IL-4 concentrations between dogs with confirmed or suspected CE and dogs with non-GI causes (*P*-value 0.264, 0.858 and 0.834, respectively, mean (standard deviation); confirmed or suspected CE group vs. non-GI causes group: IL-10 340.1 pg/mL (292.7) vs. 437.3 pg/mL (325.9), TNF-alpha 297.5 pg/mL (219.2) vs. 286.0 pg/mL (239.4), IL-4 416.8 pg/mL (449.8) vs. 445.5 pg/mL (538.8), Fig. 2). Similarly, there were no significant differences when comparing IL-10, TNF-alpha or IL-4 between dogs with only confirmed CE and non-GI causes (*P*-value 0.238, 0.785 and 0.934, respectively, mean (standard deviation); confirmed CE group vs. non-GI causes group: IL-10 324.5 pg/mL (305.3) vs. 437.3 pg/mL (325.9), TNF-alpha 267.3 pg/mL (217.5) vs. 286.0 pg/mL (239.4), IL-4 458.2 pg/mL (471.0) vs. 445.5 pg/mL (538.8).

### Comparison of IL-4:IL-10 between dogs with confirmed CE and dogs with non-GI causes

**Fig. 3:**
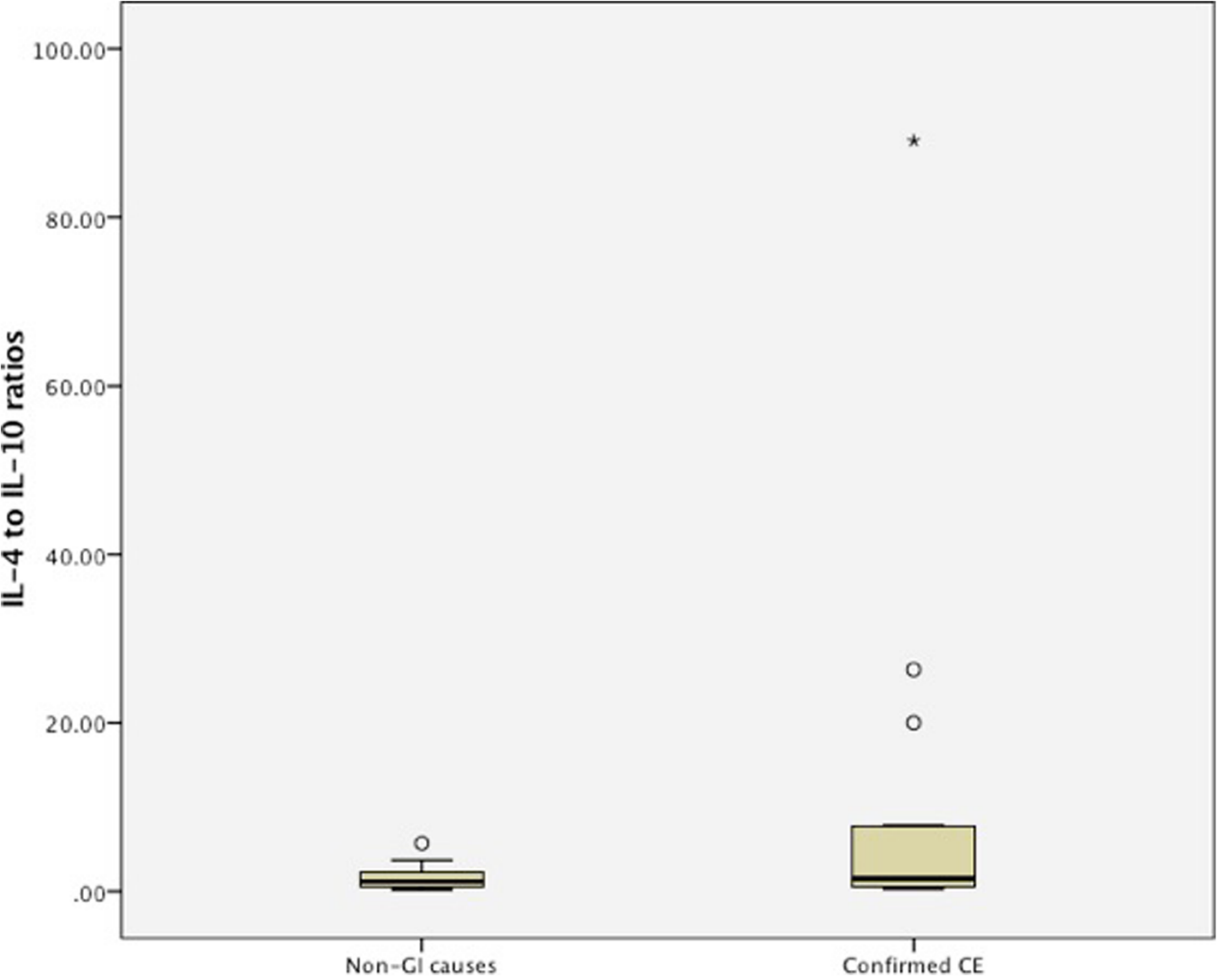
Box and whisker plot showing IL-4 to IL-10 ratios following stimulation of ex vivo whole blood with currently fed diets in dogs with CE and non-GI causes that had detectable IL-4 concentrations. Box and whisker plot of IL-4 to IL-10 ratios following stimulation of ex vivo whole blood with currently fed diets in 12 dogs with non-GI causes and 16 dogs with confirmed CE that had detectable IL-4 concentrations. There were no significant differences in IL-4:IL-10 between the two groups (*P*-value 0.147)

Independent-Samples T-tests showed no significant differences in IL-4:IL-10 between dogs with confirmed CE and detectable IL-4 concentrations and dogs with non-GI causes and detectable IL-4 concentrations (*P*-value 0.147, confirmed CE: mean 10.25, standard deviation 22.36; non-GI causes: mean 1.68, standard deviation 1.63). Six out of the 15 dogs with detectable IL-4 concentrations in the confirmed CE group had IL-4 to IL-10 ratios that exceeded the 95% confidence interval (CI) of the mean of the non-GI causes group (non-GI causes group: 95% CI of IL-4:IL-10 = 0.64–2.71; confirmed CE group: IL-4:IL-10 in 6 dogs = mean 22.40, range 2.77–89.11, Fig. 3).

### Comparison of TNF-alpha:IL-10 between dogs with confirmed CE and dogs with non-GI causes

**Fig. 4:**
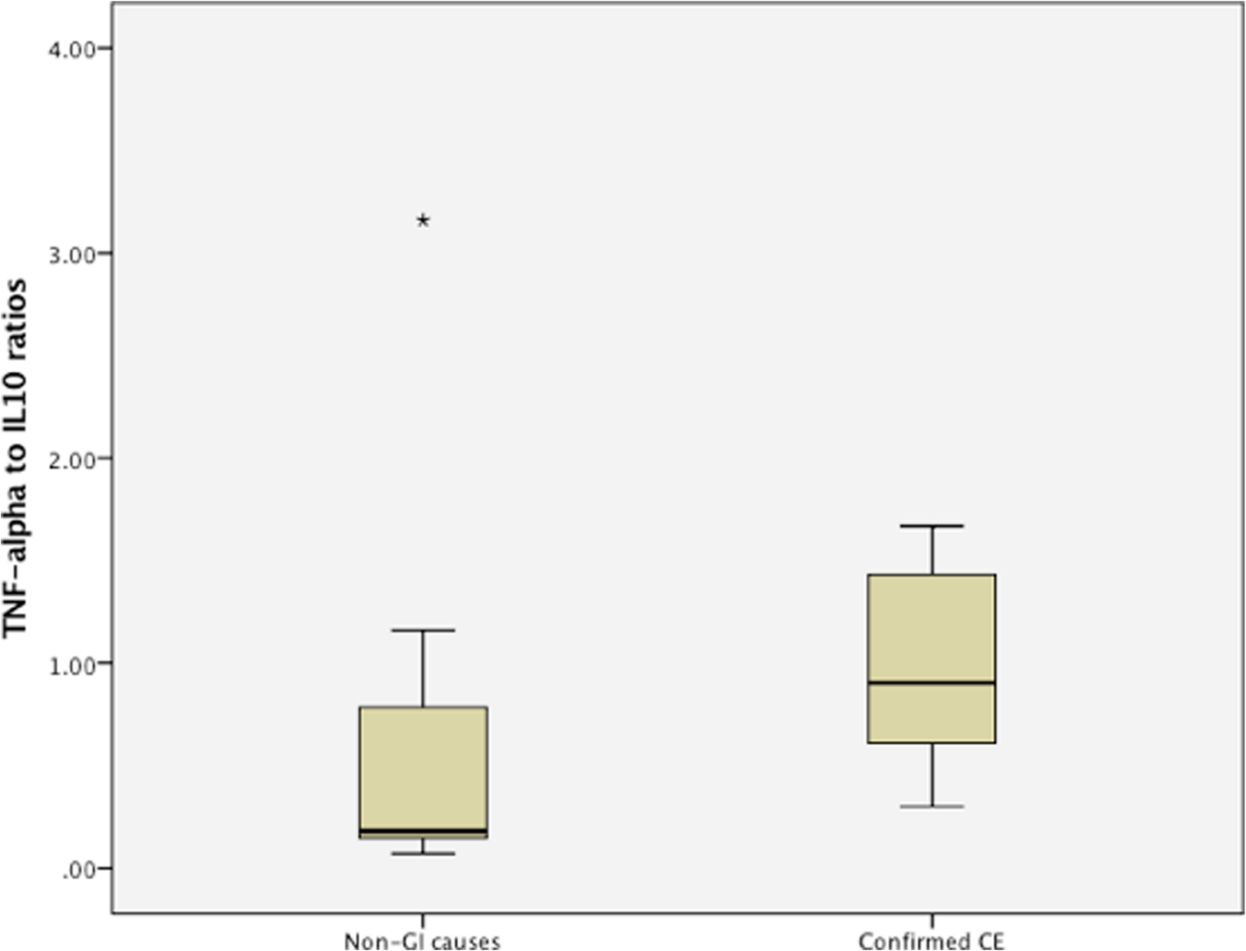
Box and whisker plot showing TNF-alpha to IL-10 ratios following stimulation of ex vivo whole blood with currently fed diets in dogs with CE and non-GI causes that had TNF-alpha concentrations within the detectable range. Box and whisker plot of TNF-alpha to IL-10 ratios following stimulation of ex vivo whole blood with currently fed diets in 7 dogs with non-GI causes and 10 dogs with confirmed CE that had TNF-alpha concentrations within the detectable range. There were no significant differences in TNF-alpha:IL-10 between the two groups (*P*-value 0.618)

Independent-Samples T-tests showed no significant differences in TNF-alpha:IL-10 between dogs with confirmed CE and TNF-alpha concentrations in the detectable range and dogs with non-GI causes and TNF-alpha concentrations in the detectable range (*P*-value 0.618, confirmed CE: mean 0.95, standard deviation 0.45; non-GI causes: mean 0.75, standard deviation 1.13). None of the 10 dogs with TNF-alpha concentrations within the detectable range in the confirmed CE group had TNF-alpha to IL-10 ratios that exceeded the 95% confidence interval (CI) of the mean of the non-GI causes group (non-GI causes group: 95% CI of TNF-alpha:IL-10 = − 0.29-1.79; Fig. 4).

## Discussion

Ex vivo whole blood stimulation assays have been previously used to characterize the cytokine response to hydrolyzed protein and commercial intact protein diets in individual, healthy, immunotolerant cats [7]. The majority of these healthy, immunotolerant cats produced detectable IL-10 concentrations but undetectable TNF-alpha and IL-4 concentrations when their ex vivo whole blood was stimulated with commercial intact protein diet that they had previously tolerated, suggesting that IL-10 may be useful as a marker for dietary tolerance [7]. Conversely, studies in humans and mice with food allergy have demonstrated increased IL-4 and TNF-alpha and decreased IL-10 concentrations [12–14, 16–19]. Therefore, our study aimed to characterize the response of these 3 cytokines to currently fed diets following incubation with ex vivo whole blood in dogs with CE and non-GI causes to help further characterize the pathogenesis of diet in CE.

Our study showed that there were no significant differences in IL-10, TNF-alpha and IL-4 concentrations between dogs with confirmed or suspected CE and dogs with non-GI causes. Although the IL-4 to IL-10 ratios were not significantly different between dogs with confirmed CE and dogs with non-GI causes, nearly half of dogs with detectable IL-4 concentrations in the confirmed CE group (approximately a quarter of all dogs with confirmed CE) had IL-4:IL-10 above the 95% confidence interval of the mean of the non-GI group. The lack of significance of any of the cytokines or their ratios between the two groups in our study may be due to the heterogeneity of disease pathogenesis in the dogs with confirmed CE. Chronic enteropathy is an idiopathic disease that is diagnosed by exclusion of all known causes of chronic GI inflammation and the confirmation of inflammatory and architectural changes on intestinal histopathology [20]. Approximately 50 to 60% of dogs with CE respond to dietary changes [21, 22]. However, most dogs with CE can be transitioned back to their original diet without showing any relapse. In one study, 8% of dogs with GI signs relapsed on challenge [2]; therefore these cases were likely food allergic or had a significant dietary risk factor for the development of their CE that triggered a relapse. Gastrointestinal food allergy can be subdivided into 2 major categories; IgE-mediated, which involves mainly mast cells and non-IgE mediated, mainly involving T-cells and eosinophils [4]. Although the inciting factors for GI food allergy are not well characterized, studies have shown the pathogenesis to be mediated by a Th2 immune response to food allergens [23]. Severe infiltration of CD4+ T cells and over production of the Th2 cytokines, including IL-4, IL-5 and IL-13 have been documented in the intestinal mucosa of humans and mouse models of GI food allergy [24, 25]. Studies have also shown increased IL-4 secreting T-cells in PBMCs isolated from humans with food allergy compared to control patients [26–28]. Some studies have also documented decreased T-regulatory cells in humans with food allergy, suggesting this may be the reason for the documented decreased IL-10 and increased IL-4 concentrations in these patients [29–32]. Although the consequences of the Th2 immune response is well characterized in food allergy, the causative factors for this response are not yet fully known. However, these patients may have a genetic risk factor, as polymorphisms in the IL-10 and IL-4 gene have been shown to have multiple associations with allergic sensitization [33]. Dogs with CE and GI food allergy may have overlapping clinical signs. Unfortunately, at this time the definitive differentiation of these two conditions in dogs is not possible. Although, the return of clinical signs following challenge with the original diet suggests food allergy, CE can not be definitively ruled out as these dogs may also relapse due to individual dietary risk factors. Therefore, the absolute proportion of dogs with confirmed or suspected CE in our study that had CE, GI food allergy or both is not known. The 6 dogs with confirmed CE and IL-4:IL-10 above the 95% CI of the mean of the non-GI group had a cytokine profile that may suggest a GI food allergy based on the studies performed in human and mouse models of GI food allergy. However, follow-up on all the dogs with CE to determine if their clinical signs completely responded to dietary management and results of any subsequent challenge trials would be needed to determine if IL-4:IL-10 could be useful to differentiate GI food allergy from CE. Similarly, treatment follow-up on all the dogs with CE may also help to determine if the cytokine response can help differentiate FRE from SRE.

Other possible reasons for the lack of significance in cytokine production between the confirmed or suspected CE and non-GI group could be due to the limited number of dogs included in each group resulting in the study being under powered. Also, the non-GI causes group contained 5 dogs with a peripheral eosinophilia at the time of sampling, which may have been an indication of an underlying food allergy, as non-GI signs can be manifestations of this disease. However, none of the dogs had current signs of cutaneous food allergy. Therefore, if these 5 dogs in the non-GI group also had a food allergy, this may have explained the lack of significance between the two groups. Another possible reason included not sampling treats that owners may have been feeding at the time of diagnosis to determine the cytokine response and therefore any adverse reactions to these additional foods would have been missed in the confirmed or suspected CE group and may have resulted in a lack of significant difference between the two groups.

Antigen primed lymphocytes circulate via the mesenteric lymph node, chyle and blood stream back to the intestinal lamina propria [34]. Therefore, our study used whole blood as a surrogate for immunological responses to diet at the intestinal lamina propria due to the circulation of these primed lymphocytes. Intact rather than enzymatically digested food was used to stimulate whole blood in our study so that proteins retained their antigenicity. Dietary proteins implicated in food allergy are those that survive or bypass digestion within the gastrointestinal tract and present intact to the immune cells in the gut [35, 36]. This is further demonstrated by the fact that antibodies to whole dietary proteins can be found in allergic as well as healthy animals [37–39]. However, whether there is an allergic reaction to the whole dietary protein depends on the class of antibody and whether there is an immune response, for example cytokine secretion or lymphocyte proliferation [40]. The intestinal mucosal surface may be able to dampen any pro-inflammatory responses to diet due to additional homeostatic immunological pathways that may be absent in the peripheral blood. This may explain why several diets in our study elicited cytokine production when incubated with ex vivo whole blood. In addition, as intact diets were incubated with whole blood, some of these intact proteins may be very easily digested in the gastrointestinal tract and therefore might never have presented intact to the immune cells within the gut in these dogs and may therefore elicit a pro-inflammatory rather than a systemic homeostatic immunological response. Therefore, confirmation of cytokine response to currently fed diets at the intestinal mucosal level is needed in dogs with CE to determine the clinical significance of our findings, as well as to confirm whether ex vivo whole blood could act as an appropriate surrogate for immunological responses to diet at the intestinal mucosal surface. Also, determining the effect of mild and moderate enzymatic digestion of the diet on cytokine response at the systemic and intestinal mucosal surface in dogs with CE may also be warranted.

Our study showed that hydrolyzed protein diets elicited significantly lower IL-10 and TNF-alpha concentrations compared to commercial intact protein diets in dogs with confirmed or suspected CE. This is similar to the cytokine response seen following stimulation of ex vivo whole blood with hydrolyzed protein diets in healthy immunotolerant cats [7]. Hydrolyzed protein diets have been successfully used in the management of CE in dogs [41, 42] and our study suggests that one mechanism for their effectiveness may be because of their ability to stimulate less cytokine production. Hydrolyzed protein diets contain peptides that are theoretically small enough to avoid an IgE meditated food allergy, by preventing cross-linking of two IgE antibody receptors on a mast cell. Although, the size of peptide needed to avoid a non-IgE mediated food allergy in dogs is currently unknown, the smaller size of the peptide in hydrolyzed protein diets is the likely reason why they elicit a reduced cytokine response. However, further studies are needed to confirm the exact mechanism of the beneficial effects of hydrolyzed protein diets in CE, as well as to confirm the cytokine response to these diets at the intestinal mucosal surface.

Other limitations of our study, in addition to the inclusion of 5 dogs with a peripheral eosinophilia in the non-GI causes group at the time of sampling and not including treats that owners may have been feeding at the time of diagnosis to determine the cytokine response include, not standardizing the lymphocyte count per well for each animal and not taking into consideration the presence of antibodies to food, although the effect of these on cytokine production to crude extracts of food is unknown. Another limitation included not definitively being able to rule out the presence of pattern recognition receptor ligands in the diet extracts, although all diet samples were processed the same way and using the same equipment including a sterile tissue culture hood and sterile filters. Also, the fact that the majority of hydrolyzed protein diets elicited undetectable pro-inflammatory cytokines following stimulation with ex vivo whole blood suggests that the technique used to process and sterilize the diets did not introduce significant bacterial contamination.

Finally, although data regarding the intra-assay and inter-assay precision, recovery and linearity of the Canine TNF-alpha and IL-10 Quantikine ELISAs with cell culture supernatants have been validated by the manufacturer, in-house validation by the authors for intra-assay and inter-assay coefficient of variation, parallelism, and spike-recoveries for all cytokine assays was not specifically performed. Therefore, another potentially serious limitation of our study is the absence of in-house validation of commercially available and widely used canine cytokine assays and therefore this could lead to inaccurate or artifactual results. However, any inaccuracies would be expected to affect all groups equally and the standard curves for all ELISAs in our study yielded an R squared value of at least 0.96, and positive and negative controls were utilized for each assay and all samples were assayed in duplicates following stimulation of triplicate wells. Nevertheless, futures studies should aim to address this limitation by ensuring that all commercial canine cytokine assays are not just validated by the manufacturer but also in-house.

## Conclusion

In conclusion, our study showed that hydrolyzed protein diets elicited a significantly reduced cytokine response when incubated with patient whole blood ex vivo compared to commercial intact protein diets in dogs with CE. As approximately a quarter of the dogs with confirmed CE had IL-4 to IL-10 ratios higher than 95% of the CI of the mean in dogs with non-GI causes, this ratio as a marker of dietary responsiveness warrants further investigation. Similarly, studies assessing the cytokine response to diet at the intestinal mucosal surface in dogs with CE are needed.

## Data Availability

Available on request from the corresponding author.

## Abbreviations

Ig: immunoglobulin
IL: interleukin
PBMC: peripheral blood mononuclear cells
PBS: phosphate buffered saline
RPMI: Roswell Park Memorial Institute
TNF: tumor necrosis factor

## Footnotes

1 50 mL Sterile Centrifuge Tubes. Corning®. Corning Incorporated, Corning, NY.

2 Minisart® NML Syringe Filter, 16,534 0.2 μm, Surfactant-free Cellulose Acetate. Sartorius Corporate Administration GmbH, Goettingen, Germany

3 Sterile culture hood, Jouan LC2.12.

4 Pierce® BCA Protein Assay Kit, ThermoFischer Scientific, Hemel Hempstead, UK.

5 PBS, Fischer, Scientific Loughborough, UK.

6 RPMI Medium 1640, ThermoFischer Scientific.

7 Penicillin-Streptomycin Solution, Sigma-Aldrich Company Ltd., Gillingham, Dorset.

8 Corning Costar®24 Well Plate, individually wrapped, sterile.

9 Sanyo CO_2_, Model MCO-15 AC.

10 Canine TNF-alpha Quantikine ELISA Kit, R&D Systems, Inc. Abingdon, UK.

11 Canine IL-10 Quantikine ELISA Kit, R&D Systems, Inc.

12 Canine IL-4 DuoSet ELISA, R&D Systems, Inc.

13 Labsystems Multiskan EX Reader.

14 Genesis Reader and Imager Software. Genesis Inc. 1998.
